# Identification of miRNAs Expression Characteristics and Biomarkers in Serum‐Derived Exosomes of Wilson’s Disease Patients

**DOI:** 10.1155/mi/9097039

**Published:** 2026-01-09

**Authors:** Hong Chen, Xie Wang, Ying Ma, Yue Pu, Hao Ye, Juan Zhang

**Affiliations:** ^1^ Department of Neurology, The First Affiliated Hospital of Anhui University of Chinese Medicine, Hefei, China, ahtcm.edu.cn; ^2^ Anhui Provincial Key Laboratory of Chinese Medicinal Formula, Hefei, China

**Keywords:** biomarkers, exosomes, miRNA, targets, Wilson’s disease

## Abstract

**Background:**

Wilson’s disease (WD), caused by mutations in the ATP7B gene, leads to copper accumulation and multi‐organ damage. Exosomal microRNAs (miRNAs) play a crucial role in cell‐to‐cell communication and the pathogenesis of diseases, yet their study in WD remains unreported. This study aims to characterize the serum exosomal miRNA signature in WD patients and investigate its potential as a source of biomarkers and therapeutic targets.

**Methods:**

Serum exosomes from WD patients and healthy controls were isolated for RNA sequencing to identify differentially expressed miRNAs (DE‐miRNAs). An integrated bioinformatics approach was employed, encompassing Gene ontology (GO), Kyoto Encyclopedia of Genes and Genomes (KEGG), Reactome, and Disease Ontology (DO) analyses to systematically decipher the functional roles, pathway involvements, and disease associations of the DE‐miRNAs. Selected DE‐miRNAs were validated by RT‐qPCR.

**Results:**

We identified 59 DE‐miRNAs (23 upregulated, 34 downregulated) in WD patient serum exosomes. GO analysis revealed their significant involvement in signal transduction, metal ion binding, and metabolic pathways. KEGG analysis highlighted alterations in key signaling cascades, including Ras, PI3K‐Akt, and Hippo pathways. Reactome analysis further uncovered disruptions in specific biological modules, notably ubiquitin‐mediated proteolysis, GPCR signaling, and spliceosome assembly. DO enrichment demonstrated significant associations with hepatocellular carcinoma, neuropsychiatric disorders, and metabolic diseases. RT‐qPCR validation confirmed the reliability of DE‐miRNA expression patterns (*p*  < 0.05).

**Conclusions:**

This study establishes the first comprehensive landscape of serum exosomal miRNAs in WD, revealing their involvement in an interconnected network of pathological processes. Our findings provide a novel conceptual framework for understanding WD pathophysiology and pinpoint promising candidates for biomarker development.

## 1. Introduction

Wilson disease (WD), an autosomal recessive disorder caused by pathogenic variants in the ATP7B gene, is characterized by abnormal copper accumulation in the liver, brain, and other organs, leading to hepatic dysfunction, neurological manifestations, and multisystemic complications [1]. Current diagnostic approaches primarily rely on serum ceruloplasmin measurement, 24‐h urinary copper excretion, and molecular genetic analysis [2]; however, these methodologies exhibit limitations in early disease detection, subtype classification, and therapeutic response monitoring [3, 4]. The identification of novel biomarkers for WD holds significant potential to advance noninvasive diagnosis, targeted therapy development, and treatment efficacy evaluation.

Exosomes are nano‐sized vesicles secreted by cells, with diameters ranging from ~50 to 150 nm [5]. They can carry bioactive molecules such as proteins, nucleic acids, and lipids [6]. Exosomes have a unique mode of action; they interact with recipient cells through endocytosis, binding ligands to receptors, allowing the integrated materials to be transferred between cells [7]. This process plays a crucial role in cell‐to‐cell communication and the development of diseases. MicroRNAs (miRNAs), small endogenous noncoding RNAs, can regulate the expression of target proteins by acting on mRNA [8]. Studies have found that exosomes released by human macrophages, NK cells, and other cells contain various types of RNA, including mRNA and miRNA [9, 10]. These RNAs are delivered to the corresponding recipient cells, where they exert specific functions [11].

An increasing number of studies have demonstrated that exosomal miRNAs, as crucial regulatory molecules, exhibit disease‐specific expression profiles in various neurological disorders and liver diseases, positioning them as potential diagnostic biomarkers and therapeutic targets. Research has shown that serum and exosomal miR‐7‐1‐5p and miR‐223‐3p may serve as biomarkers for Parkinson’s disease [12]. Furthermore, the expression profile of serum‐derived exosomal miRNAs can be utilized to assess the severity of non‐alcoholic fatty liver disease (NAFLD), aiding in the identification of potential therapeutic targets for NAFLD treatment [13]. Additionally, it has been found that exosomal miRNA‐342‐3p derived from primary hepatic macrophages promotes liver fibrosis by inhibiting HPCAL1 in stellate cells [14]. However, the precise role of exosomal miRNAs in WD remains unreported. This study focuses on exosomal miRNAs in the serum of patients with WD, employing high‐throughput RNA sequencing technology to thoroughly analyze differential gene expression patterns. Based on these findings, we aim to accurately identify specific biomarkers of serum miRNAs for WD, thereby providing novel insights and a robust scientific foundation for the diagnosis and treatment of this condition.

## 2. Materials and Methods

### 2.1. Participants

This study primarily enrolled 15 patients with WD who met the criteria and received treatment in the inpatient department of the Brain Disease Center at the First Affiliated Hospital of Anhui University of Chinese Medicine from January 2024 to December 2024. Additionally, 15 healthy volunteers were included as controls.

### 2.2. Diagnostic Criteria

#### 2.2.1. Criteria for Diagnosing WD

In accordance with the clinical diagnostic criteria for WD outlined in the “Chinese Guidelines for the Diagnosis and Treatment of Hepatolenticular Degeneration (2021 Edition)” [15], the possibility of Wilson’s disease (WD) should be considered in patients presenting with unexplained liver manifestations, neurological symptoms (particularly extrapyramidal symptoms), or psychiatric symptoms. The age of onset should not be used as a basis for diagnosing or excluding WD. The specific diagnostic points are recommended as follows:

1. Neurological and/or psychiatric symptoms.
2. Unexplained liver damage.
3. Reduced serum ceruloplasmin and/or elevated 24‐h urinary copper.
4. Positive corneal K‐F rings.
5. Both chromosomes of the patient carry pathogenic variants of the ATP7B gene, as determined by familial co‐segregation and pathogenicity analysis of genetic variations.

A definitive diagnosis of WD can be made when criteria (1 or 2) + (3 and 4) or (1 or 2) + 5 are met. If criteria 3 + 4 or 5 are met without obvious clinical symptoms, the individual is diagnosed as a presymptomatic carrier of WD. If any two of the first three criteria are met, the condition is diagnosed as “possible Wilson’s disease,” and further follow‐up observation is recommended, including ATP7B gene testing for a definitive diagnosis.

#### 2.2.2. Inclusion Criteria

WD patients included in the study should meet the following criteria: (1) All enrolled patients with WD in this study met the diagnostic criteria for “definitely diagnosed cases” as specified in the “Chinese Guidelines for the Diagnosis and Treatment of Hepatolenticular Degeneration (2021 Edition).” Patients at the presymptomatic stage or meeting criteria for probable cases were excluded, thereby ensuring homogeneity of the study cohort and reliability of the results and (2) both the patient and their family members agree to participate in this research and have signed the informed consent form.

#### 2.2.3. Exclusion Criteria

The exclusion criteria are as follows: (1) individuals aged below 10 years or above 50 years; (2) pregnant or lactating women; (3) those with severe mental or behavioral abnormalities; (4) individuals with comorbid viral hepatitis, autoimmune hepatitis, alcoholic liver disease, or organic brain diseases such as brain tumors, encephalitis, epilepsy, and traumatic brain injuries; and (5) patients with severe conditions, including hepatic encephalopathy, hepatorenal syndrome, upper gastrointestinal bleeding, portal vein thrombosis, and other unstable medical conditions.

### 2.3. Collection of Demographic and Clinical Data and Blood Samples

Demographic and clinical data, including name, age, and gender, were collected from both the control group (CG) and patients with WD (WDG). From all enrolled cases, three pairs of blood samples (A1‐A3, D1‐D3) were randomly selected for the extraction of serum exosomal vesicles. Additionally, 12 pairs of blood samples were utilized for the validation of sequencing data.

### 2.4. Isolation, Extraction, and Identification of Exosomes

Exosomes were extracted using ultracentrifugation. After thawing the samples at 37°C, they were transferred to a new centrifuge tube and centrifuged at 2000×*g* for 30 min at 4°C. The supernatant was then moved to a fresh tube and centrifuged again at 10,000×*g* for 45 min at 4°C to remove larger vesicles. The resulting supernatant was filtered through a 0.45‐μm filter membrane, and the filtrate was collected. This filtrate was transferred to another new tube and subjected to ultracentrifugation at 100,000×*g* for 70 min at 4°C using an ultracentrifugal rotor. The supernatant was discarded, and the pellet was resuspended in 10 mL of pre‐chilled 1× PBS. Ultracentrifugation was performed once more under the same conditions (100,000×*g*, 70 min, 4°C). After removing the supernatant again, the pellet was resuspended in 150 μL of pre‐chilled 1× PBS. The sample was divided into two groups; from each group, 20 μL was taken and mixed to form one sample for electron microscopy. Additionally, 20 μL was used for particle size analysis, 10 μL for fluorescence analysis, and the remaining exosomes were stored at −80°C.

Transmission electron microscopy (TEM) was employed to observe the extracted exosomes. A 10 μL aliquot of the exosome sample was deposited onto a copper grid and allowed to settle for 1 min, followed by removal of excess liquid with filter paper. Subsequently, 10 μL of uranyl acetate was applied to the grid, left to incubate for 1 min, and then the surplus solution was wicked away using filter paper. After air‐drying at room temperature for several minutes, the grid was examined under TEM at an accelerating voltage of 80 kV to obtain images.

For particle size analysis of the extracted exosomes, a 10 μL volume of the exosome suspension was diluted to an appropriate concentration. Following confirmation of instrument performance through calibration with standards, the diluted exosome samples were loaded onto the device. Gradual dilution steps were implemented to prevent clogging of the injection needle. Upon completion of the measurement, the instrument provided data on both the size distribution and concentration of the exosomes.

Nano‐flow cytometry was used to detect the expression of fluorescent markers on exosomes. Twenty microliters of exosomes were diluted to 60 microliters, and 30 microliters of the diluted exosomes were separately mixed with 20 microliters of fluorescently labeled antibodies (CD9, CD81). The mixtures were incubated at 37°C for 30 min in the dark. One milliliter of pre‐chilled PBS was then added, and ultracentrifugation was performed at 4°C and 110,000×*g* for 70 min using a high‐speed rotor. The supernatant was removed, and another 1 ml of pre‐chilled PBS was added before repeating the ultracentrifugation step under the same conditions. The supernatant was again removed, and the pellet was resuspended in 50 μL of pre‐chilled 1× PBS. Instrument performance was tested using standards before loading the exosome samples, which required gradient dilution to prevent clogging of the injection needle. The protein indicator results from the samples were obtained once the detection was completed.

### 2.5. Total RNA Extraction and Quality Control

Exosome RNA was isolated and purified using the exoRNeasy Midi/Maxi Kit (QIAGEN, Germany), followed by assessment of RNA integrity using the 300/5400 Fragment Analyzer (Agilent, CA, USA). The detailed procedures are as follows: (1) Exosome Purification: Pre‐filtered serum (excluding particles larger than 0.8 μM) was mixed with Buffer XBP and loaded onto an exoEasay affinity membrane spin column for binding. Vesicles bound to the membrane were washed with Buffer XWP, followed by lysis with QIAzol. (2) Total RNA Isolation: Chloroform was added to the QIAzol eluate, and the aqueous phase was recovered and mixed with ethanol. Total RNA, including miRNA, was bound to the spin column, washed three times, and then eluted.

### 2.6. Library Construction and miRNA Sequencing and Identification

The experimental procedures were conducted following the standard protocols provided by Illumina, encompassing library preparation and sequencing experiments. The sRNA sequencing library was prepared utilizing the TruSeq Small RNA Sample Prep Kits (Illumina, San Diego, USA). Upon completion of the library preparation, the constructed libraries were sequenced using the Illumina HiSeq 2000/2500 platform, with a single‐end read length of 50 base pairs. Initially, raw data were filtered to process the obtained reads and generate clean reads. Subsequently, sRNA sequences were filtered based on their length, retaining those within the range of 18–26 nucleotides. The remaining sequences were aligned against various RNA databases, including mRNA, Rfam, and Repbase (excluding miRNA), to filter out non‐miRNA sequences and obtain valid data. The valid data were then compared against the human mature and precursor sequences in the miRBase 22.0 database, as well as the genomic sequences of the species under study, for the identification of known and novel miRNAs. The expression levels of miRNAs in each sample were calculated from the length‐filtered data using ACGT101‐miR, and statistical normalization methods were applied to the expression values.

### 2.7. Differential Expression Analysis of miRNAs With Functional and Pathway Enrichment Assessment Using Gene Ontology (GO), Kyoto Encyclopedia of Genes and Genomes (KEGG), Reactome, and Disease Ontology (DO) Databases

The input data consisted of normalized values (norm), with *p*‐values calculated using a model based on the normal distribution. A significance threshold of *p*  < 0.05 was applied to identify differentially expressed miRNAs (DE‐miRNAs). Subsequent analyses included clustered heatmaps, volcano plots, and scatter plots to visualize the data. To systematically investigate the functional roles and regulatory mechanisms of DE‐miRNAs, we conducted an integrated bioinformatics analysis utilizing multiple databases. GO analysis categorized the biological functions of DE‐miRNA targets across molecular functions (MFs), cellular components (CCs), and biological processes (BPs). KEGG enrichment analysis identified significantly impacted signaling cascades and metabolic pathways, defining their regulatory roles within core networks. Reactome analysis provided deeper insights into dynamic processes governed by DE‐miRNAs, including cell signaling, metabolic flux, and gene expression regulation. Importantly, DO analysis revealed associations between DE‐miRNA signatures and specific diseases. These findings collectively established a crucial link between DE‐miRNA and WD pathogenesis, elucidating their significant contribution to disease development and progression.

### 2.8. Prediction and Construction of the miRNA Target Gene Network

Based on the differential expression analysis of miRNAs and the prediction of the relationships between miRNAs and their target genes, the Cytoscape v3.10.1 software (http://www.cytoscape.org/) was utilized to construct the miRNA‐target interaction network. This was done to further evaluate the interactions between miRNAs and their target genes.

### 2.9. RT‐qPCR Validation

RT‐qPCR technology was employed to detect the expression of the identified DE‐miRNAs. Initially, total RNA was meticulously extracted from the samples and then subjected to a reverse transcription process to convert it into the corresponding cDNA. Subsequently, specific primers were selected for PCR amplification of different types of cDNA. In this process, two microliters of the cDNA template were precisely added to each PCR reaction system to ensure consistent reaction conditions. Thereafter, the 2−*ΔΔ*CT method was utilized for data processing. The CT value of the target gene was carefully compared with the CT value of *β*‐actin, which served as the endogenous control. The difference between the two values was used to rigorously estimate the relative expression level of the target gene, thereby achieving a reliable quantitative assessment of the expression level of DE‐miRNAs.

### 2.10. Statistical Analysis

All data in this study were subjected to statistical analysis using SPSS 27.0 software. For comparing two independent sets of numerical variables, an independent samples *t*‐test was employed when the data conformed to a normal distribution. In instances where the assumptions of normality, homogeneity of variances, or independence were not met, non‐parametric tests were utilized. The chi‐square test (*X* ^2^) was applied to analyze the correlation and differences in completely randomized categorical variables. A *p*‐value less than 0.05 was considered statistically significant. The Benjamini–Hochberg procedure was adopted to adjust for multiple comparisons in the GO and KEGG enrichment analyses, with a significance threshold of *p* < 0.05 indicating substantial enrichment of the respective terms or pathways.

## 3. Results

### 3.1. Comparison of General Information

A total of 30 participants, including patients with WD and healthy controls, were included in this study. Demographic and baseline characteristics were comparable between the two groups, with no significant differences observed in age, sex, or basic diseases such as diabetes, hyperlipidemia, and hypertension (*p*  > 0.05). Other clinical parameters, including clinical typing, diagnostic parameters, serum liver function, neurological function, and psychiatric symptom assessments, are summarized in Table 1. Further visual representation of the data comparisons and distributions is provided in (Figure 1A–J).

Figure 1General Information Statistics. (A) Comparison of age between two groups (ns indicated no statistical significance). (B–D) Comparison of liver function between two groups. (E,F) Comparison of serum ceruloplasmin and 24‐h urine copper between two groups. (G–J) Comparison of gender and basic disease composition between two groups.

**Figure:**
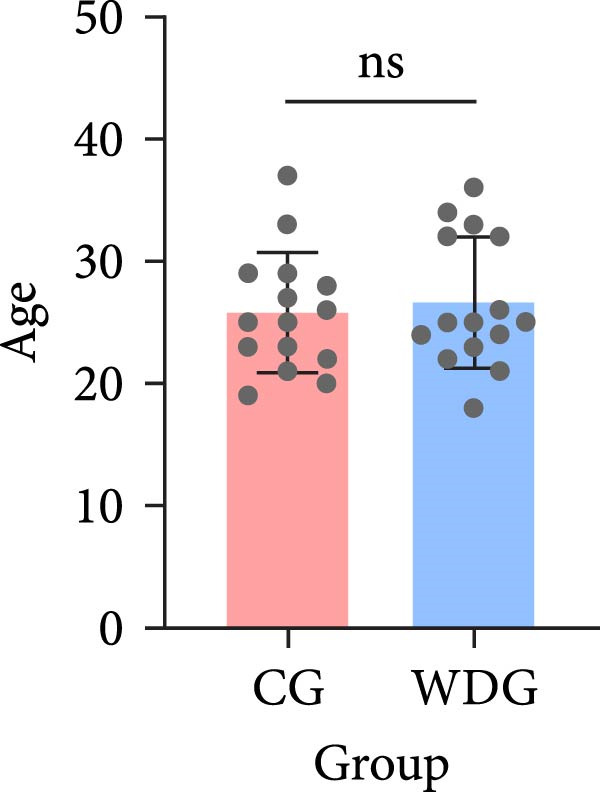
(A)

**Figure:**
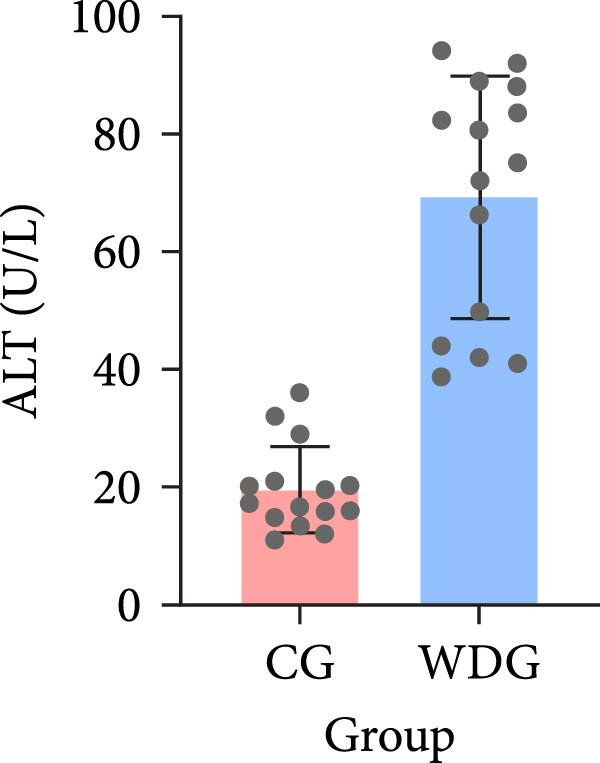
(B)

**Figure:**
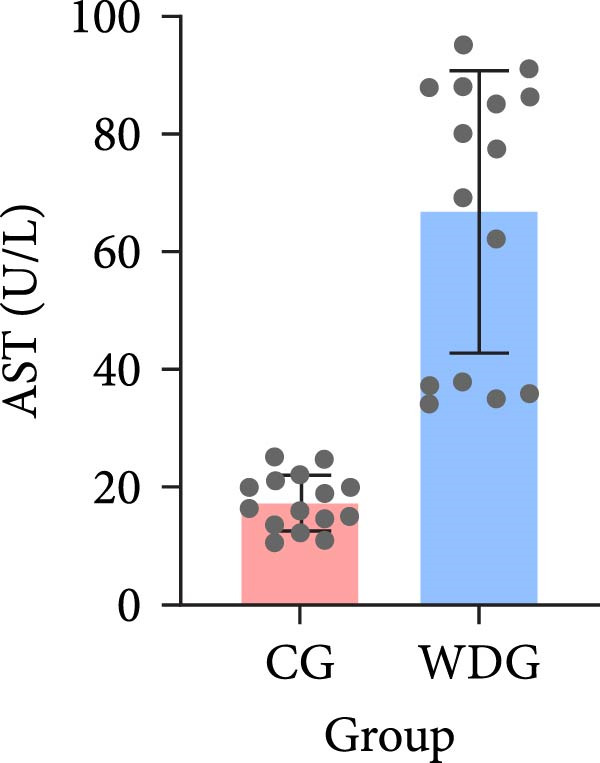
(C)

**Figure:**
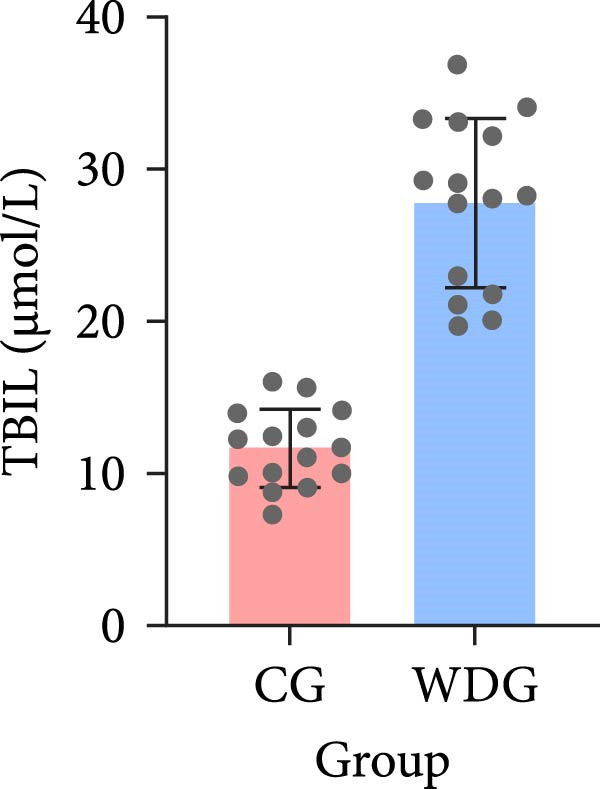
(D)

**Figure:**
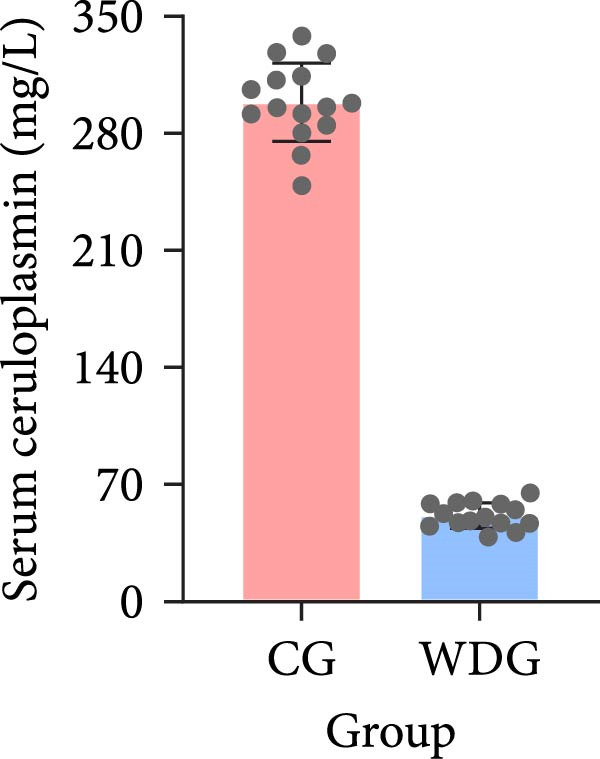
(E)

**Figure:**
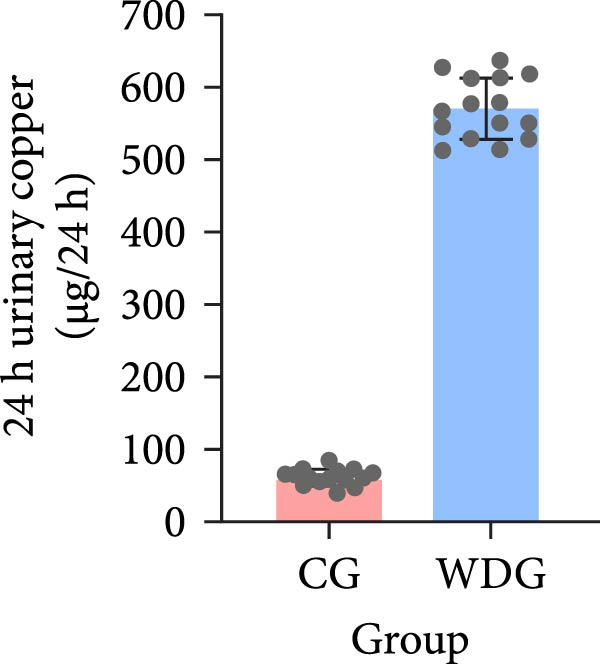
(F)

**Figure:**
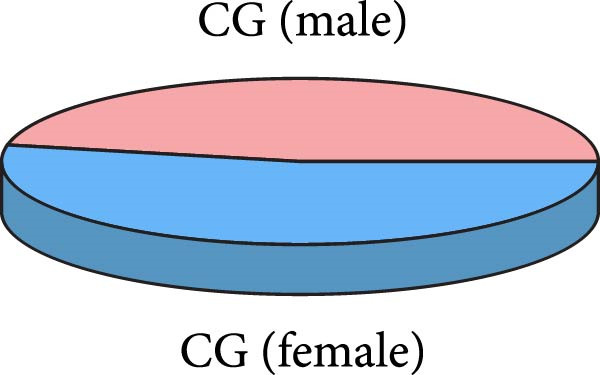
(G)

**Figure:**
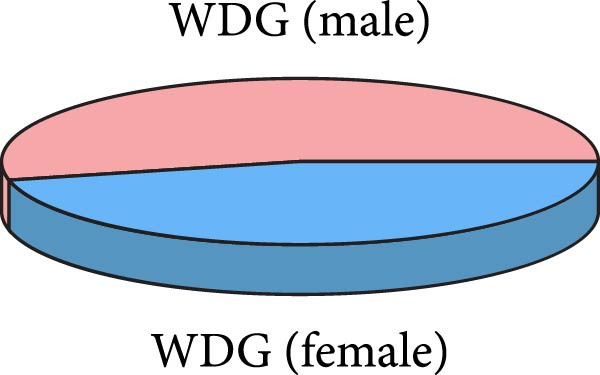
(H)

**Figure:**
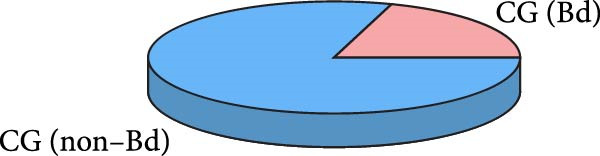
(I)

**Figure:**
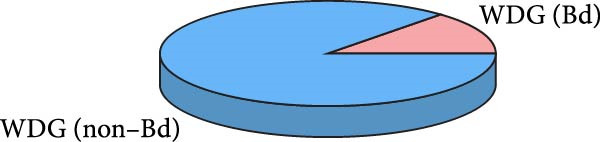
(J)

**Table 1:** Statistics of general information.

Characteristic	WDG (*n* = 15)	CG (*n* = 15)	*p*‐Value
Demographics			
Age (years), mean ± SD	26.67 ± 5.37	25.80 ± 4.92	0.648
Gender (Male, %)	8 (53.3%)	7 (46.7%)	0.715
Clinical typing/*n* (%)			
Hepatic	7 (46.7%)	—	—
Neurological	5 (33.3%)	—	—
Mixed (hepatic and neurological)	3 (20.0%)	—	—
Diagnostic parameters			
Serum ceruloplasmin (mg/L), mean ± SD	50.71 ± 15.51	298.13 ± 23.64	<0.001
24‐h urinary copper (μg/24 h), mean ± SD	518.36 ± 10.45	62.28 ± 11.27	<0.001
K‐F rings positive/*n* (%)	10 (66.7%)	N/A	—
Detected ATP7B mutations/*n* (%)	14 (93.33%)	N/A	—
Liver Function Indexes			
ALT (U/L), mean ± SD	69.25 ± 20.57	19.66 ± 7.32	<0.001
AST (U/L), mean ± SD	66.81 ± 23.96	17.43 ± 4.68	<0.001
TBIL (μmol/L), mean ± SD	27.76 ± 5.56	11.65 ± 2.57	<0.001
Neurological function and psychiatric symptom scoring (UWDRS‐Ⅰ/‐Ⅲ, mean ± SD)	16.33 ± 9.4/6.2 ± 3.51 (neurological and mixed type patients)	—	—
Basic diseases, *n* (%)	2 (13.33%)	3 (20.00%)	0.547

### 3.2. Observation and Identification of Exosome Morphology and Characteristics

Under TEM, exosomes in CG appeared predominantly round with clear boundaries. In contrast, those isolated from WDG exhibited irregular morphology and heterogeneous size distribution (Figure 2A,B). The mean diameter of vesicles in the CG group was ~94.2 nm, with a concentration of 5.47 × 10^9^ particles/mL; corresponding values for the WDG group were 88.6 nm and 1.19 × 10^9^ particles/mL, respectively (Figure 2C–F). Analysis via nanoscale flow cytometry revealed reduced expression levels of the surface marker proteins CD9 and CD81 on exosomes derived from WDG compared to those from CG (Figure 2G–L).

Figure 2Observation and identification of exosomes. (A) Morphological characteristics of exosomes in CG under TEM; (B,C) Analysis of exosomes size and concentration of CG (D–F) Expression of exosomes marker proteins CD9 and CD81 in CG. (G) Morphological characteristics of exosomes of WDG under TEM; (H,I) Analysis of exosomes size and concentration of WDG (J–L) Expression of exosomes marker proteins CD9 and CD81 of WDG.

**Figure:**
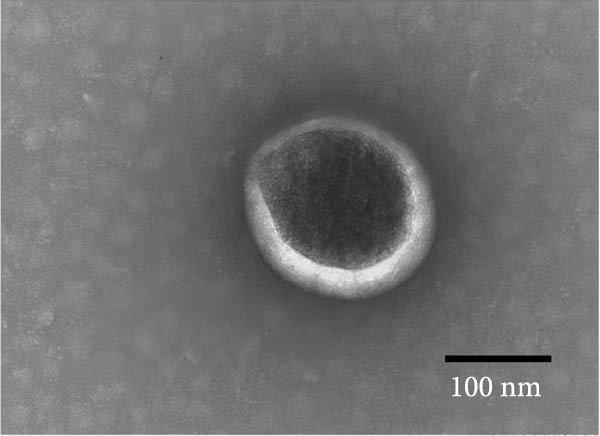
(A)

**Figure:**
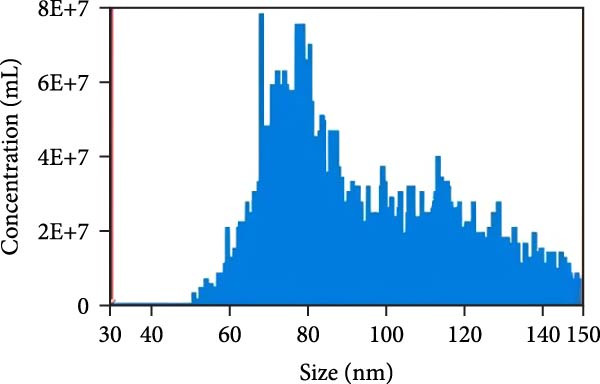
(B)

**Figure:**
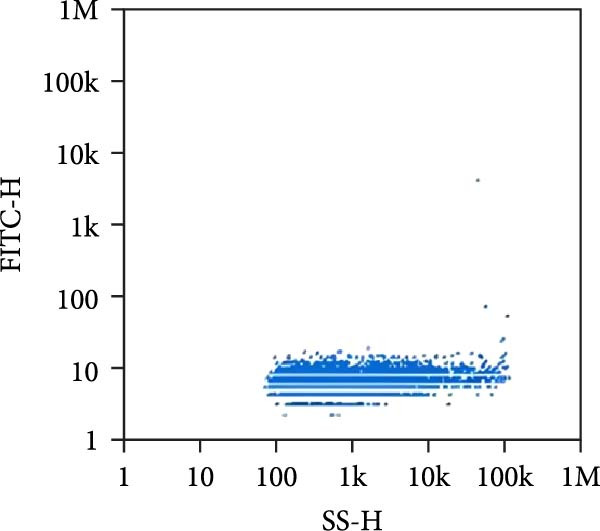
(C)

**Figure:**
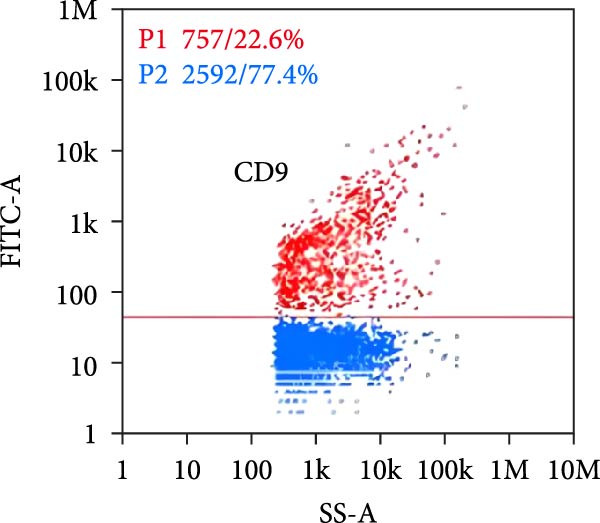
(D)

**Figure:**
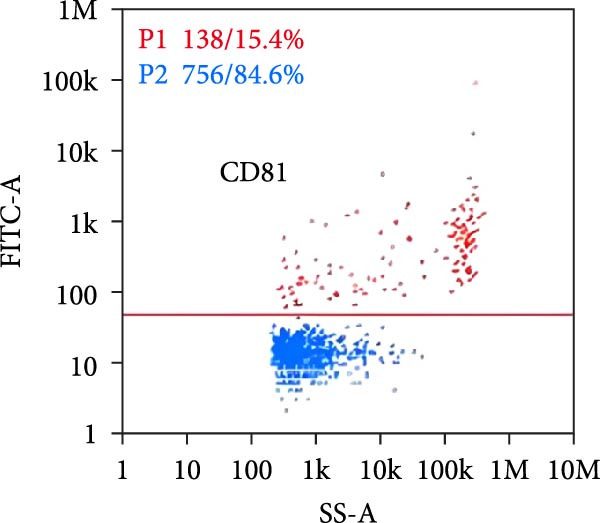
(E)

**Figure:**
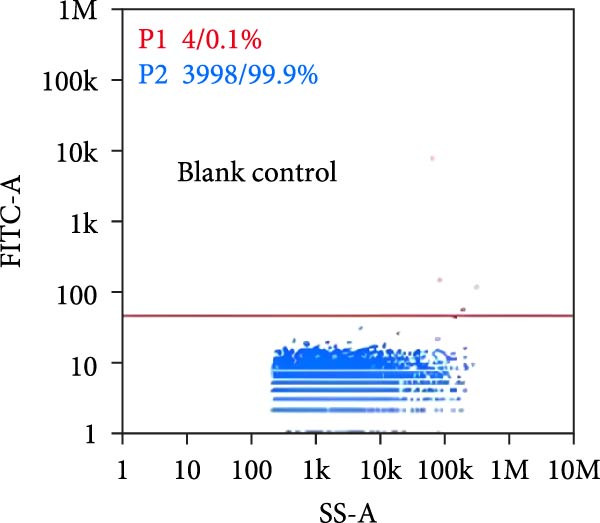
(F)

**Figure:**
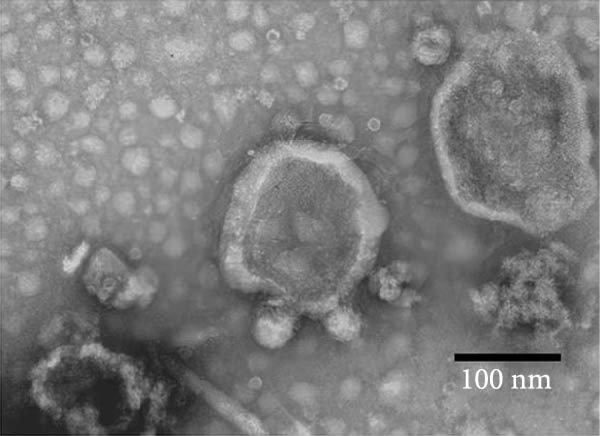
(G)

**Figure:**
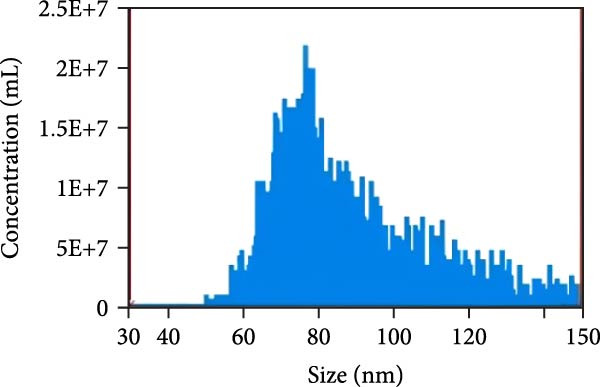
(H)

**Figure:**
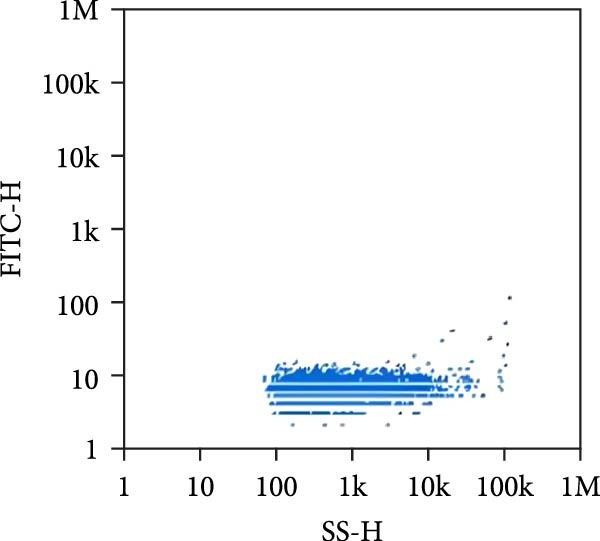
(I)

**Figure:**
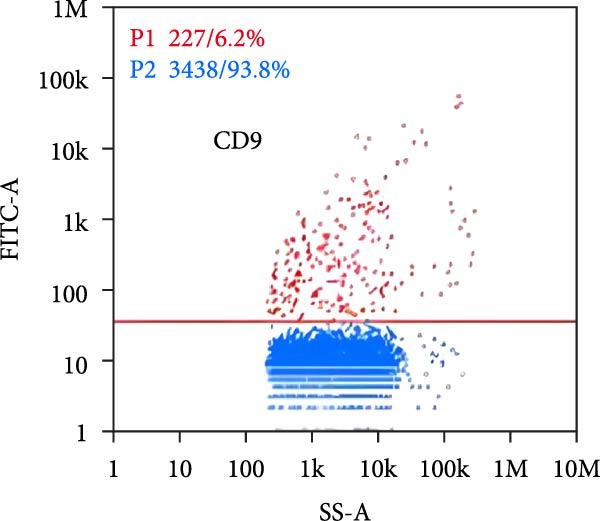
(J)

**Figure:**
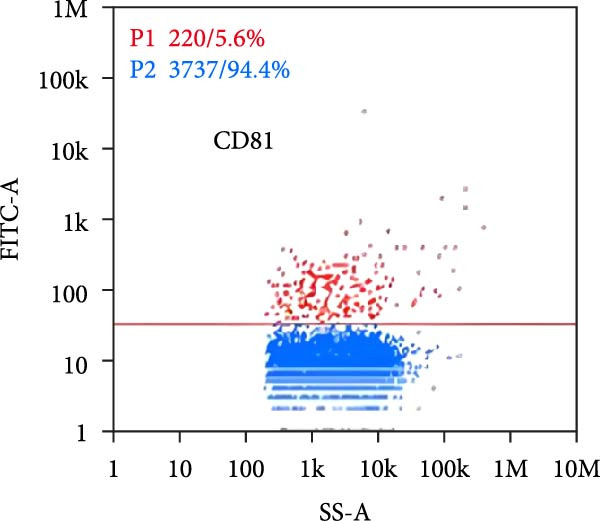
(K)

**Figure:**
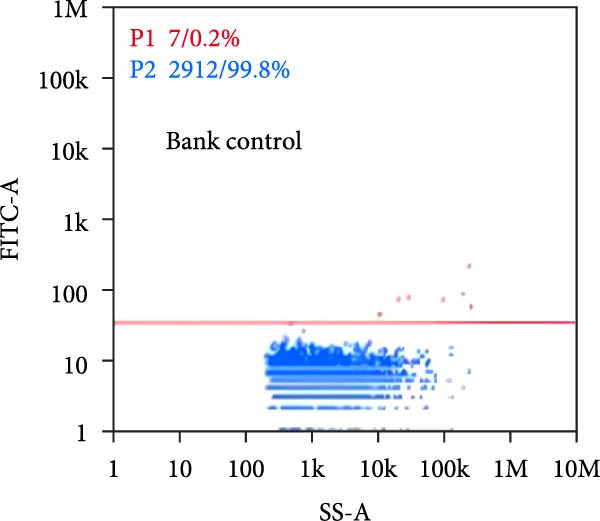
(L)

### 3.3. Quantitative Analysis of miRNA Expression Between Two Groups

A total of 1364 miRNAs were identified in the study, with 893 commonly expressed in both the CG and WDG groups. Additionally, 199 miRNAs were predominantly expressed in the CG group, while 272 exhibited specific expression in the WDG group (Figure 3A). Notably, the detected miRNAs were distributed across all chromosomes except chr4, chr8, chr10, chr16, and chrY, showing varying degrees of correlation with miRNA expression patterns (Figure 3B,C).

Figure 3Quantitative analysis of miRNA expression in two groups. (A) The Venn diagram of miRNA expression in two groups. (B) The distribution of miRNA expression chromosomes in CG. (C) The distribution of miRNA expression chromosomes in WDG.

**Figure:**
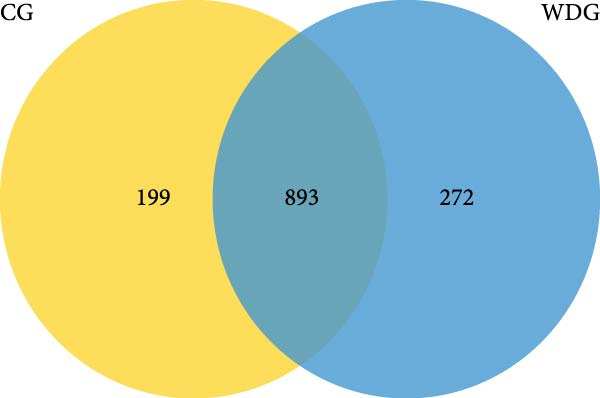
(A)

**Figure:**
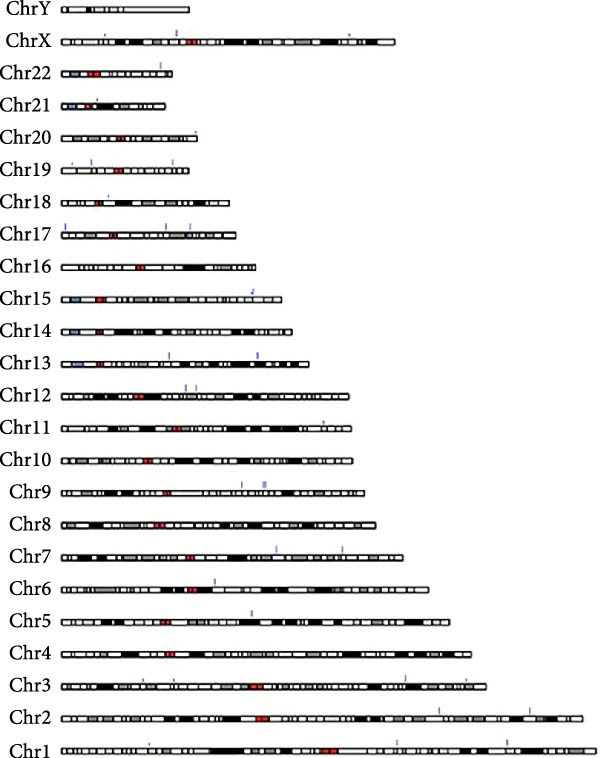
(B)

**Figure:**
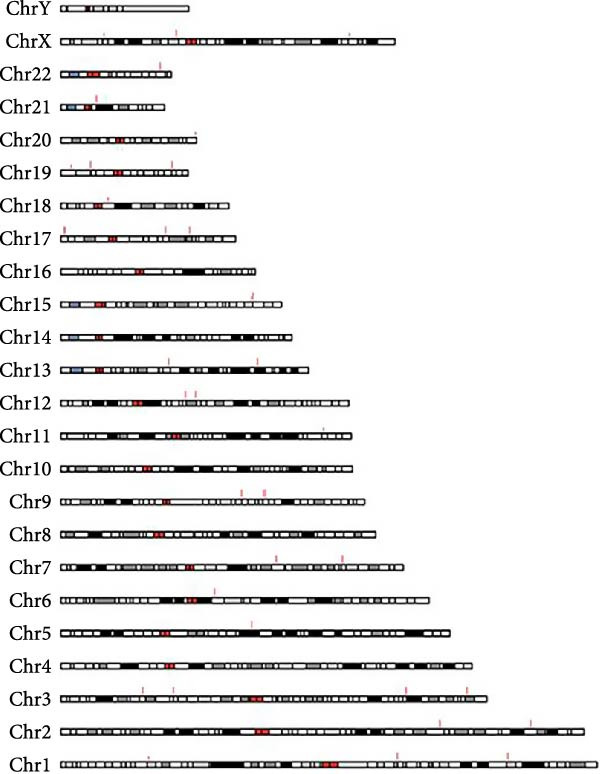
(C)

### 3.4. Identification of DE‐MiRNAs in Serum‐Derived Exosomes From WD Patients

Under the screening criteria of |log2(fold change)| ≥ 1 and *p*‑value  < 0.05, this study identified a total of 59 DE‐miRNAs in serum exosomes from WD patients. Specifically, among the identified DE‐miRNAs, 23 were significantly up‐regulated, and 34 were significantly down‐regulated. Notably, four of the downregulated exosomal miRNAs exhibited a *p*‑value  < 0.01. The top 10 known DE‐miRNAs are listed in Table 2. Principal component analysis (PCA), volcano plots, scatter plots, and heatmaps were generated to illustrate the distribution characteristics of the identified DE‐miRNAs (Figure 4A–D).

Figure 4Expression and distribution characteristics of DE‐miRNA in serum derived exosomes from CG and WDG. (A) Principal component analysis chart. (B) DE‐miRNA expression volcano plot. (C) Scatter plot of DE‐miRNA expression distribution. (D) DE‐miRNA distribution heatmap.

**Figure:**
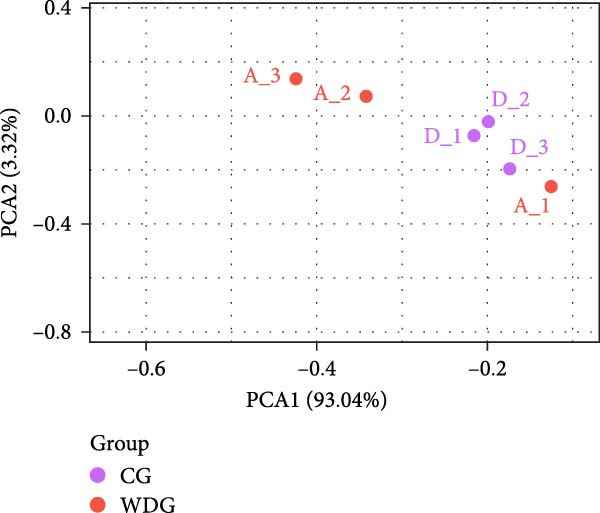
(A)

**Figure:**
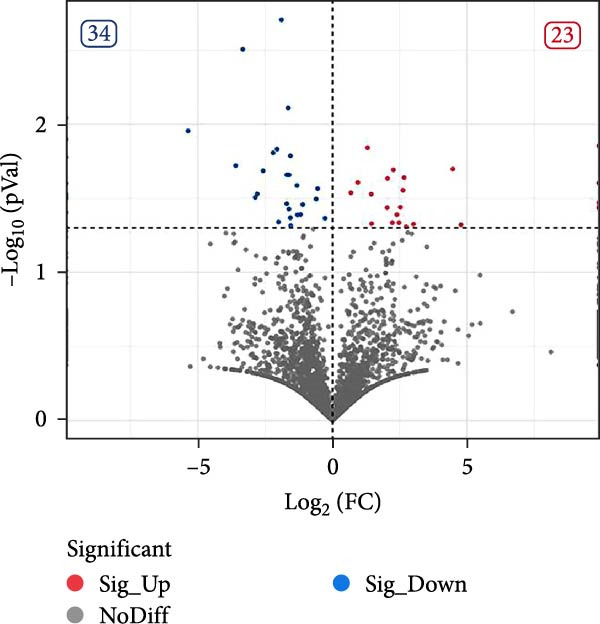
(B)

**Figure:**
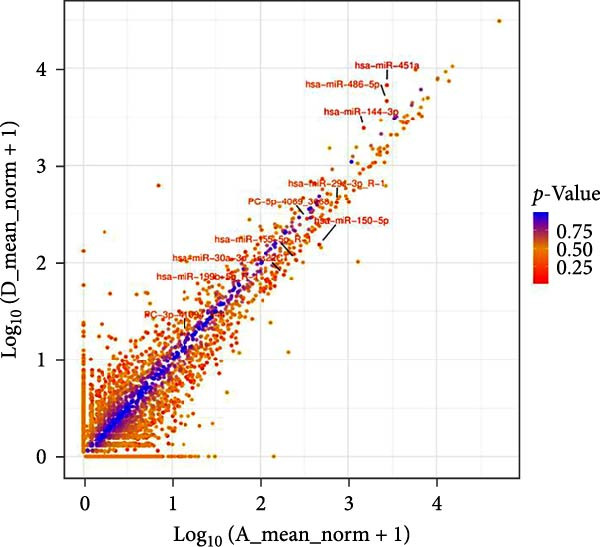
(C)

**Figure:**
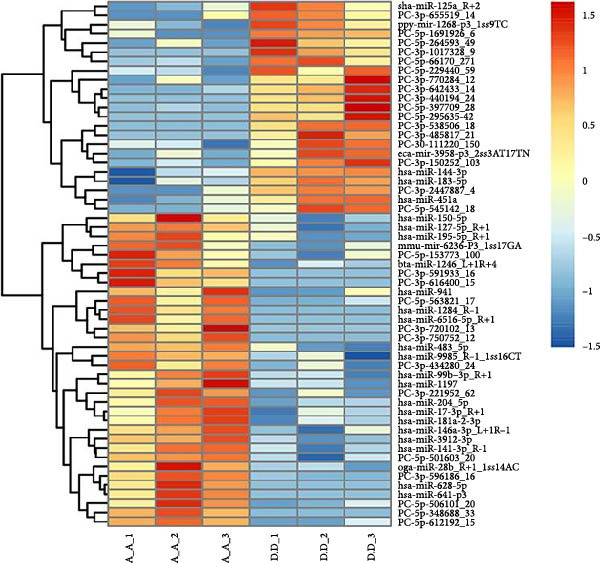
(D)

**Table 2:** Detailed information on the top 10 DE‐miRNAs in serum‐derived exosomes from patients with WD.

miR_name	miR_seq	log2FC	*p*‐Value	Up/down
hsa‐miR‐3912‐3p	TAACGCATAATATGGACATGT	−1.89	0.00	Down
hsa‐miR‐141‐3p_R‐1	TAACACTGTCTGGTAAAGATG	−1.64	0.01	Down
hsa‐miR‐451a	AAACCGTTACCATTACTGAGTT	1.31	0.01	Up
hsa‐miR‐146a‐3p_L+1R‐1	ACCTCTGAAATTCAGTTCTTCA	−1.56	0.02	Down
hsa‐miR‐127‐5p_R+1	CTGAAGCTCAGAGGGCTCTGATT	−1.31	0.03	Down
hsa‐miR‐204‐5p	TTCCCTTTGTCATCCTATGCCT	−2.86	0.03	Down
hsa‐miR‐483‐5p	AAGACGGGAGGAAAGAAGGGAG	−1.69	0.03	Down
sha‐miR‐125a_R+2	TCCCTGAGACCCTAACTTGTGAAA	2.40	0.04	Up
hsa‐miR‐150‐5p	TCTCCCAACCCTTGTACCAGTG	−1.61	0.04	Down
hsa‐miR‐941	CACCCGGCTGTGTGCACATGTGC	−1.18	0.04	Down

### 3.5. GO Analysis of DE‐MiRNAs Derived From Serum Exosomes of WD Patients

Through GO analysis of the expression of DE‐miRNAs in exosomes derived from the serum of WD patients, a total of 50 significantly enriched BPs and functional items were identified. These items consisted of 25 BP terms, 15 CC terms, and 10 MF terms. Among the BP terms were signal transduction (GO:0007165), regulation of transcription by RNA polymerase II (GO:0006357), regulation of DNA‐templated transcription (GO:0006355), positive regulation of transcription by RNA polymerase II (GO:0045944), and cell differentiation (GO:0030154). The CC terms included membrane (GO:0016020), cytoplasm (GO:0005737), nucleus (GO:0005634), cytosol (GO:0005829), and another membrane term (GO:0016021). The MF terms encompassed protein binding (GO:0005515), metal ion binding (GO:0046872), DNA binding (GO:0003677), transferase activity (GO:0016740), and nucleotide binding (GO:0000166). The results of the GO analysis for the expressed DE‐miRNAs, along with the top 20 enriched GO terms, were presented in Figure 5A–D.

Figure 5GO analysis of DE‐miRNAs expression in serum‐derived exosomes of WD patients. (A) A summary barplot of GO enrichment classification. (B) TOP20 BP classification enrichment chart. (C) TOP20 CC classification enrichment chart. (D) TOP20 MF classification enrichment chart.

**Figure:**
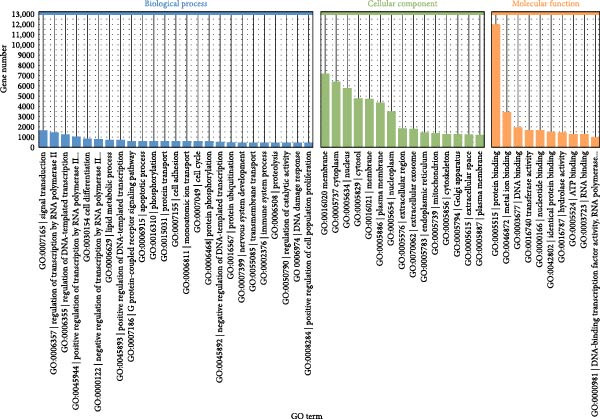
(A)

**Figure:**
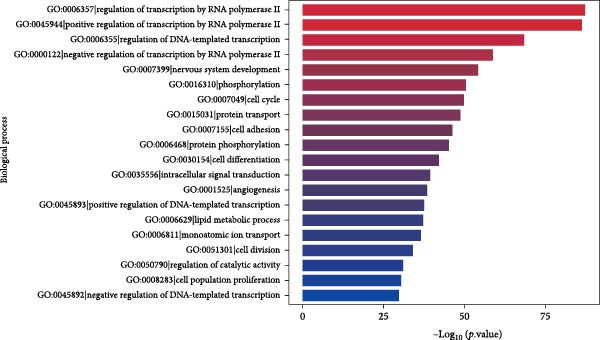
(B)

**Figure:**
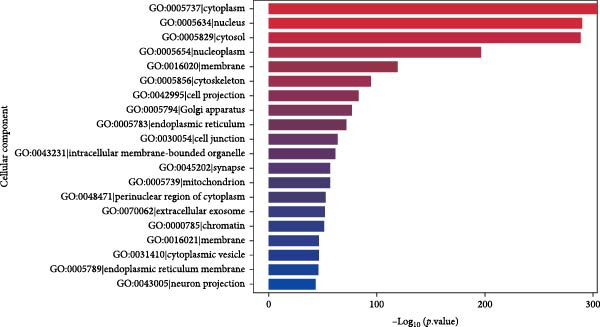
(C)

**Figure:**
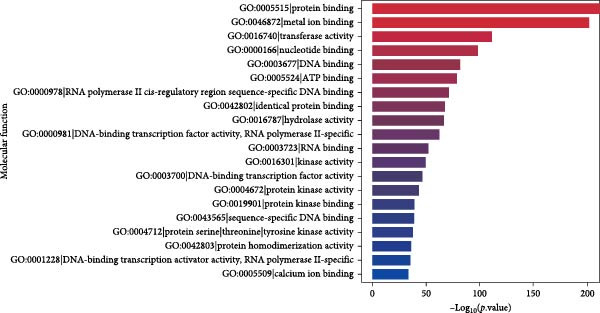
(D)

### 3.6. KEGG Analysis of DE‐miRNAs Derived From Serum Exosomes of WD Patients

To further elucidate the enrichment of DE‐miRNAs in biological pathways, KEGG analysis was employed. The results revealed that the identified DE‐miRNAs were significantly enriched in six major categories, including cellular processes, environmental information processing, genetic information processing, human diseases, metabolism, and organism systems, encompassing a total of 30 relevant biological pathways. Notably, the top enriched pathways included metabolic pathways, pathways in cancer, Rap1 signaling pathway, axon guidance, Ras signaling pathway, calcium signaling pathway, regulation of actin cytoskeleton, Hippo signaling pathway, proteoglycans in cancer, PI3K‐Akt signaling pathway, focal adhesion, ubiquitin‐mediated proteolysis, hepatocellular carcinoma, oxytocin signaling pathway, glutamatergic synapse, and ErbB signaling pathway. Figure 6A–C illustrates the categorized summary chart, the top 20 enriched pathways diagram, and the bubble plot, respectively, based on the KEGG analysis outcomes.

Figure 6KEGG, Reactome and DO analysis of DE‐miRNAs expression in serum‐derived exosomes of WD patients. (A) A summary barplot of KEGG enrichment classification. (B) TOP20 of KEGG enrichment chart. (C) KEGG enrichment scattor plot. (D) Reactome enrichment analysis chart; (E) DO enrichment analysis chart.

**Figure:**
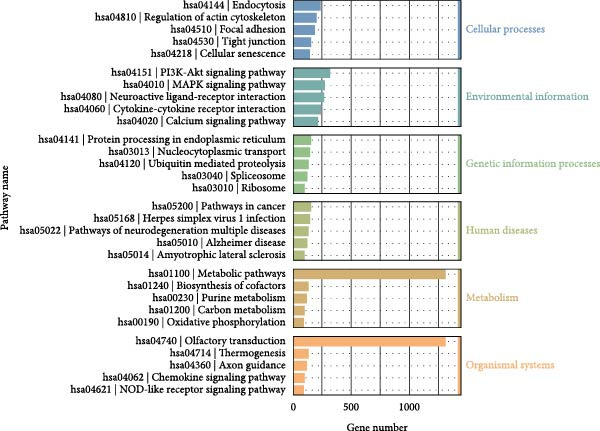
(A)

**Figure:**
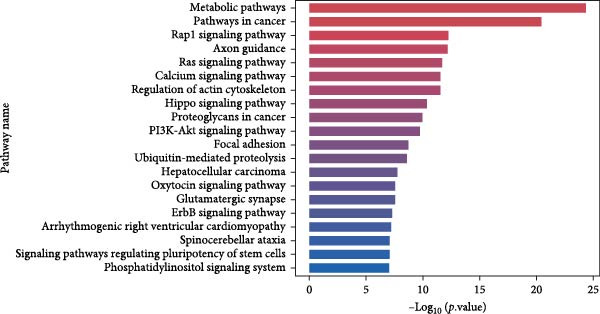
(B)

**Figure:**
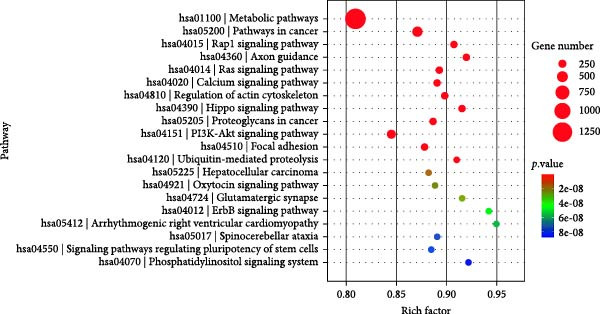
(C)

**Figure:**
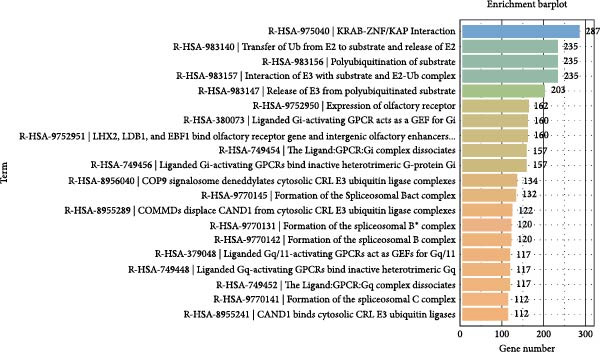
(D)

**Figure:**
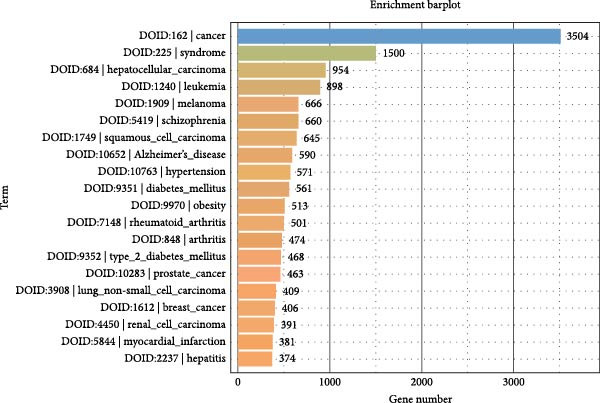
(E)

### 3.7. Reactome and DO Analysis of DE‐miRNAs Derived From Serum Exosomes of WD Patients

The Reactome analysis revealed significant enrichment of these DE‐miRNAs in specific biological pathways, including those involved in genetic regulation and gene silencing (KRAB‐ZNF/KAP interaction), ubiquitination processes such as “Transfer of Ub from E2 to substrate and release of E2,” “Polyubiquitination of substrate” “Interaction of E3 with substrate and E2‐Ub complex,” “Release of E3 from polyubiquitinated substrate”; G protein‐coupled receptor signaling pathways like “Liganded Gi‐activating GPCR acts as a GEF for Gi”; spliceosome assembly events including “Formation of the Spliceosomal Bact complex” and “Formation of the Spliceosomal B complex”; and olfactory function‐related pathways such as “Expression of Olfactory Receptor” (Figure 6D). DO analysis demonstrated substantial enrichment across diverse disease categories: prominent cancers (hepatocellular carcinoma, leukemia, melanoma, squamous cell carcinoma, prostate cancer, non‐small cell lung cancer, breast cancer, and renal cell carcinoma); neuropsychiatric disorders (schizophrenia, Alzheimer’s disease); metabolic and immune conditions (hypertension, diabetes mellitus, obesity, and rheumatoid arthritis); and other diseases including myocardial infarction and hepatitis (Figure 6E).

### 3.8. Prediction of DE‐miRNA Targets and Analysis of Target Network Interactions

**Figure 8:**
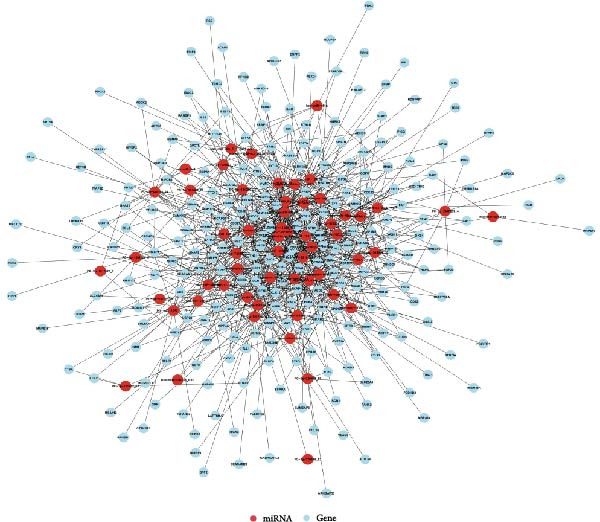
Interaction network diagram between TOP 1000 DE‐miRNA and predicted targets.

To further elucidate the roles of exosomal‐derived DE‐miRNAs and their corresponding target genes in WD, we conducted a predictive analysis of the targeted relationships among the identified genes, with select results summarized in Table 3. Building upon these predictions, we proceeded to construct interaction networks for the top 100, 500, and 1000 DE‐miRNA targets, as depicted in Figures 7A,B, and 8.

Figure 7Interaction network diagram between TOP 100, 500 DE‐miRNA and predicted targets. (A) Interactions between TOP 100 DE‐miRNA and predicted targets. (B) Interactions between TOP 500 DE‐miRNA and predicted targets.

**Figure:**
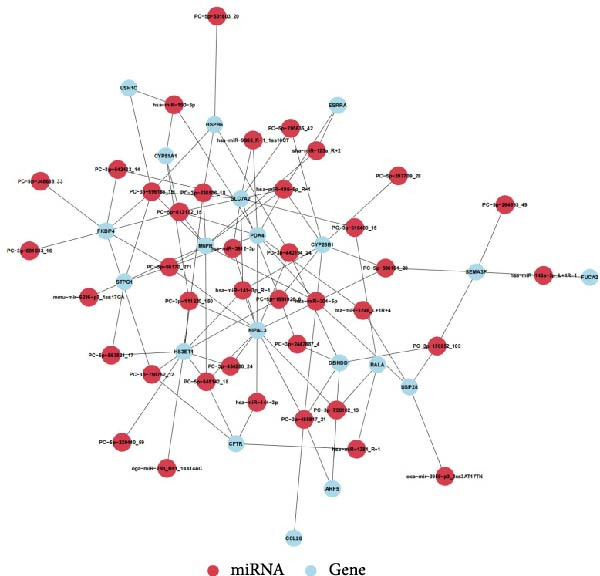
(A)

**Figure:**
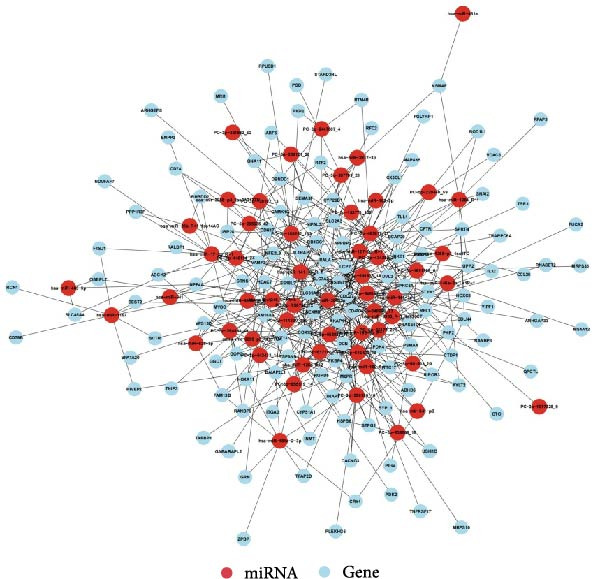
(B)

**Table 3:** Data on partially predicted miRNA‐target gene interactions.

miRNA.ID	Transcript.ID	Gene.ID	Target gene	TargetScan.score
PC‐5p‐2372428_4	ENST00000000233	ENSG00000004059	ARF5	61
PC‐5p‐6237779_2	ENST00000000233	ENSG00000004059	ARF5	89
hsa‐miR‐301a‐5p	ENST00000000412	ENSG00000003056	M6PR	94
PC‐5p‐586292_16	ENST00000000442	ENSG00000173153	ESRRA	75
PC‐3p‐354125_32	ENST00000001008	ENSG00000004478	FKBP4	94
PC‐3p‐642433_14	ENST00000001008	ENSG00000004478	FKBP4	70
PC‐3p‐485817_21	ENST00000001146	ENSG00000003137	CYP26B1	50
PC‐5p‐1691926_6	ENST00000001146	ENSG00000003137	CYP26B1	71
PC‐5p‐295635_42	ENST00000001146	ENSG00000003137	CYP26B1	66
hsa‐miR‐17‐3p_R+1	ENST00000002125	ENSG00000003509	NDUFAF7	94

### 3.9. RT‐qPCR Validation

Six DE‐miRNAs identified through preliminary screening, including the upregulated hsa‐miR‐183‐5p, hsa‐miR‐451a, and hsa‐miR‐144‐3p, as well as the downregulated hsa‐miR‐204‐5p, hsa‐miR‐483‐5p, and hsa‐miR‐150‐5p, were randomly selected for further validation at the gene expression level using RT‐qPCR on the remaining samples from both groups. The results demonstrated statistically significant differences in the expression of these miRNAs between the CG and the WDG (*p* *＜*0.05), indicating the reliability of the detection outcomes (Figure 9).

Figure 9(A–F) Barplot for RT‐qPCR validation of selected DE‐miRNA ( ^∗^ *p*  < 0.05).

**Figure:**
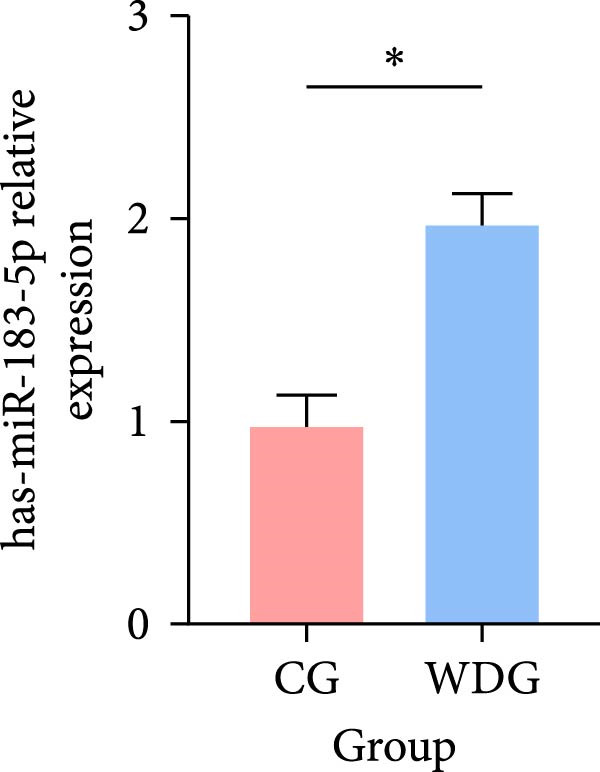
(A)

**Figure:**
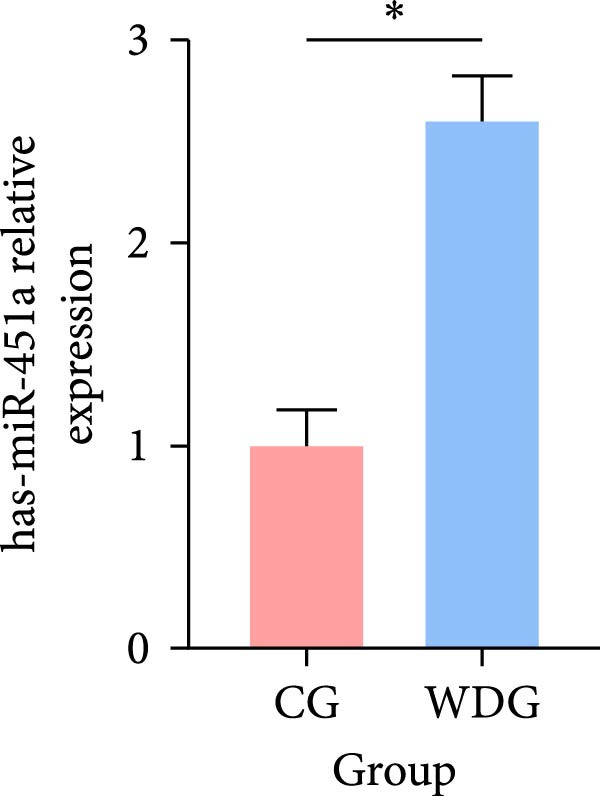
(B)

**Figure:**
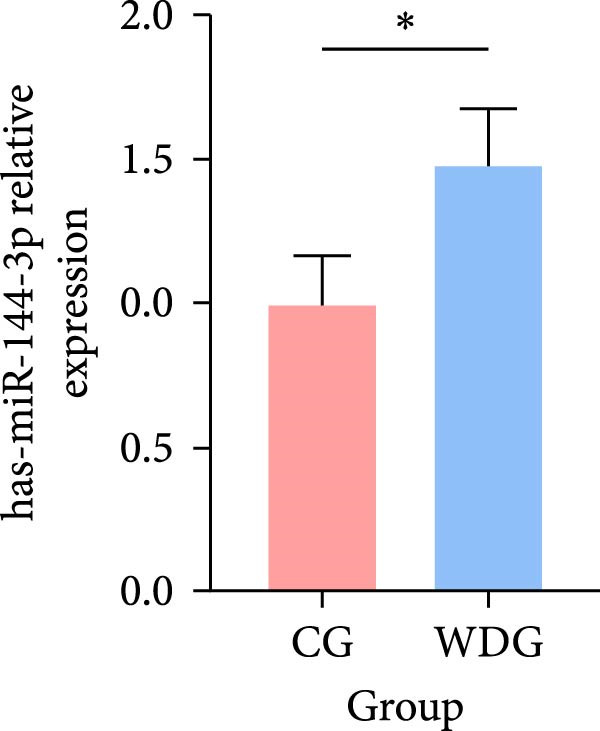
(C)

**Figure:**
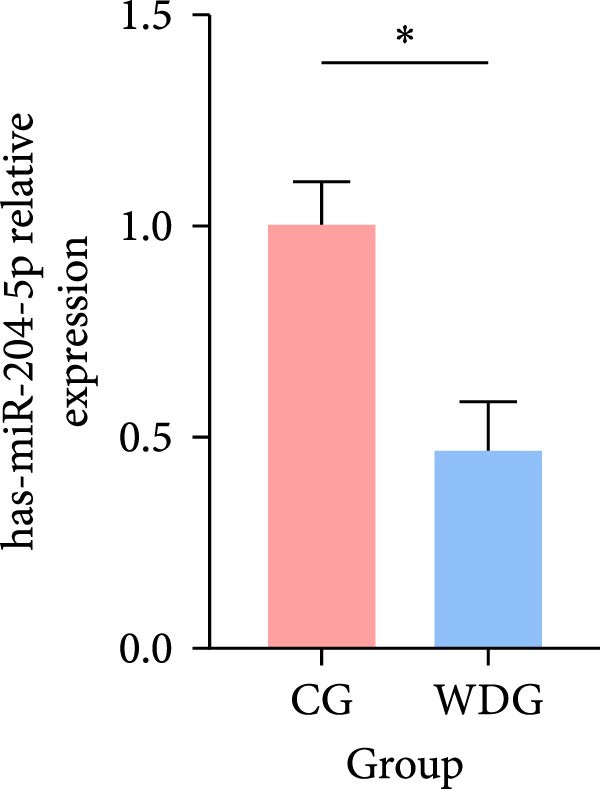
(D)

**Figure:**
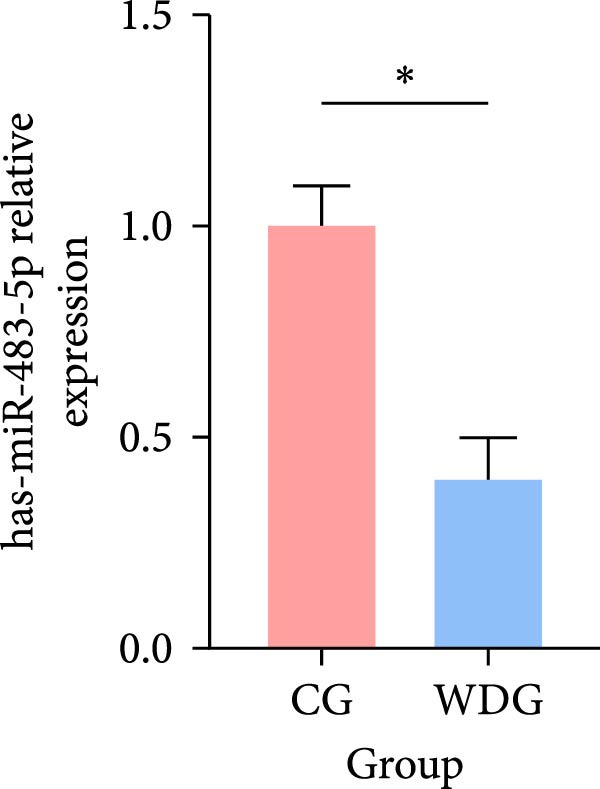
(E)

**Figure:**
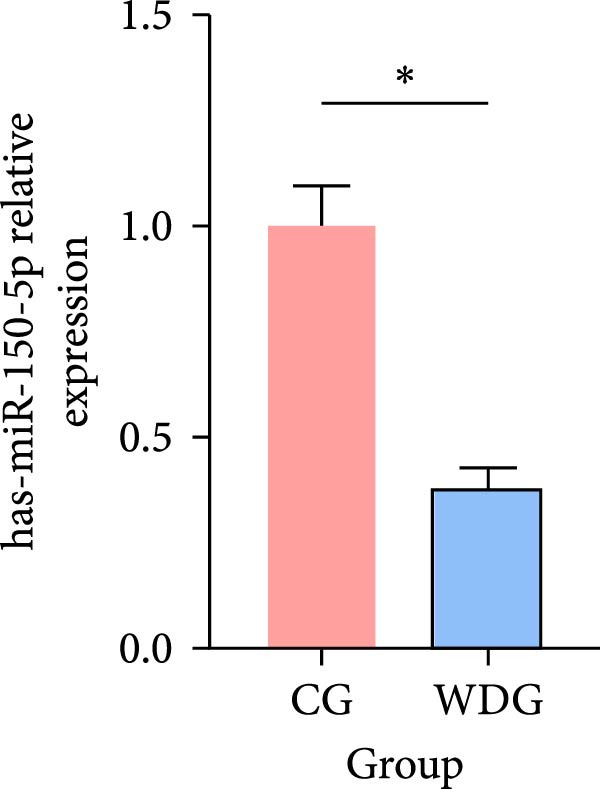
(F)

## 4. Discussion

Conventional pathological understanding of WD centers on copper toxicity resulting from ATP7B gene mutations and subsequent metabolic dysfunction. However, the clinical heterogeneity observed in patients suggests the involvement of broader molecular network disruptions. Although exosome‐derived miRNAs serve as crucial mediators of intercellular communication [16], their global expression profiles and functional roles in WD remain largely unexplored. Employing high‐throughput sequencing, this study pioneered the characterization of serum exosomal miRNA expression profiles in individuals with WD, identifying 59 DE‐miRNAs (23 upregulated and 36 downregulated). Through comprehensive bioinformatics analyses coupled with RT‐qPCR validation of selected candidates, we provide novel insights into the complex pathogenesis of WD from the perspective of noncoding RNA regulation.

Among the DE‐miRNAs identified in our study, miR‐141‐3p demonstrates significant functional relevance to WD pathology through its regulatory roles in both hepatic and neurological processes. Research shows that downregulation of miR‐141‐3p promotes hepatic stellate cell activation and liver fibrosis progression via the PTEN/AKT signaling pathway [17]. In neurological contexts, miR‐141‐3p participates in regulating neural stem cell neurogenesis [18] and modulates neuronal apoptosis by targeting PBX1 to regulate PROK2 transcription in cerebral ischemia models [19]. Additionally, this miRNA has been shown to inhibit the proliferation and differentiation of bone marrow‐derived mesenchymal stem cells [20]. These multifaceted functions position miR‐141‐3p as a potentially crucial regulator connecting WD’s characteristic hepatic and neurological pathologies through its effects on HSCs and neural stem cells.

Similarly, miR‐483‐5p emerges as a dual player in hepatic fibrosis and neuroprotection. Elevated levels of miR‐483‐5p/3p have been shown to collaboratively impede liver fibrosis in transgenic mice subjected to transforming growth factor (TGF) stimulation [21]. This microRNA also manifests antiproliferative properties against hepatocellular carcinoma cells, concomitantly reducing cellular steatosis and fibrosis by targeting PPAR*α* and TIMP2 [22]. Its neuroprotective action against mitochondrial dysfunction triggered by cardiac arrest, mediated via the TNFSF8/AMPK/JNK signaling cascade, is well documented [23].

The inclusion of miR‐204‐5p in our analysis draws attention to its relevance in liver cancer development. Studies confirm its regulatory influence over hepatocellular carcinoma progression through SIRT1 modulation [24] and its intimate link with hepatoblastoma via angiogenic control mediated by the JAK2/STAT3 pathway [25].

Regarding miR‐451a, it earns recognition for its substantial impact on select liver disorders. It mitigates alcoholic hepatitis by curbing hdac8‐driven inflammatory responses [26] and alleviates hepatic steatosis while inhibiting hepatitis C virus replication through glycerol kinase targeting [27], implying a dual modulatory role in inflammation and metabolism within WD contexts.

Moreover, miR‐125a‐5p demonstrates significant ties to metabolic derangements, notably ameliorating glycolipid metabolic abnormalities in type 2 diabetes mellitus through STAT3 modulation [28]. Perturbed miR‐125a expression is also implicated in enhanced angiogenesis via increased glycolysis [29], indicative of a central role in hepatic metabolic homeostasis during WD development.

As for miR‐146a‐3p, it is identified as an ERBB2 inhibitor, obstructing the differentiation of human adipose‐derived mesenchymal stem cells (hAMSCs) into Schwann‐like cells [30]; however, the detailed mechanisms of this miRNA’s interaction with neural cells in WD require further elucidation.

Research on miR‐127‐5p primarily centers on bone‐related pathologies and traumatic brain injury [31, 32], while miR‐150‐5p has been implicated in both neoplasms and renal fibrosis [33–35]. The functional significance of miR‐3912 and miR‐941 in WD remains largely unexplored, though their aberrant expression has been documented in various malignancies [36–38], warranting further investigation into their potential roles.

In this study, GO analysis revealed that target genes of DE‐miRNAs were significantly enriched in fundamental BPs such as signal transduction, transcriptional regulation, and metal ion binding. Notably, the enrichment of the “metal ion binding” function directly corresponds to a core feature of WD pathophysiology—copper metabolic dysfunction. Furthermore, extensive involvement in transcriptional and differentiational regulation suggests that exosomal miRNAs may modulate target cell functional states through epigenetic mechanisms. Subsequent KEGG pathway analysis demonstrated widespread participation of DE‐miRNAs in metabolic and oncogenic pathways (e.g., Ras/PI3K‐Akt [39]), directly linking WD‐associated metabolic abnormalities to hepatocellular carcinoma risk. Dysregulation of neural pathways (e.g., axon guidance, glutamatergic synapse) provides a molecular basis for neurological symptoms observed in WD patients. Disruptions in Hippo signaling pathway [40], regulation of actin cytoskeleton [41], and focal adhesion [42] potentially mediate impaired liver fibrosis and tissue repair processes in WD, collectively elucidating its multisystem pathological mechanisms. Moreover, Reactome analysis uncovered dysregulation at finer resolution levels of cellular functional modules. Most prominently, significant enrichment was observed within the ubiquitin‐proteasome system, implying disrupted protein homeostasis as a novel pathogenic node in WD. Concurrently, enrichment of G protein‐coupled receptor signaling pathways indicates potential alterations in cellular responses to extracellular stimuli including hormones and neurotransmitters [43]. Additionally, spliceosome assembly pathway enrichment suggests exosomal miRNAs from WD may influence precursor mRNA splicing events [44], thereby contributing to disease progression.

Finally, DO enrichment analysis robustly corroborated the clinical relevance of these findings. Target genes of the DE‐miRNAs showed significant associations with diverse diseases, including hepatocellular carcinoma, schizophrenia, Alzheimer’s disease, diabetes mellitus, and rheumatoid arthritis. Notably, profound enrichment for cancers suggests that a pro‐tumorigenic microenvironment may exist within WD patients. Enrichment for neuropsychiatric disorders independently supports—and provides novel research directions for—the comorbidity of psychiatric symptoms, behavioral abnormalities, and cognitive impairment in individuals with WD. Furthermore, the observed enrichment for metabolic and immune diseases not only underscores WD as a systemic condition impacting whole‐body metabolism and immune status but also indicates an elevated risk of comorbidities in affected individuals. Taken together, our results suggest that exosomal miRNAs derived from WD patients may disrupt multiple core BPs—ranging from protein homeostasis and signal transduction to gene expression regulation—thereby collectively forming the molecular basis underlying its multisystem clinical manifestations.

This study has several limitations. First, this study utilized Chinese diagnostic guidelines, which are aligned with international standards in principle; nonetheless, future multicenter and multinational studies that directly adopt internationally recognized criteria will be crucial to validate our findings and enhance their generalizability. Moreover, the identified differential gene expressions still require validation through in vivo and in vitro experiments. Additionally, the research methods employed in this study are relatively singular; therefore, integrating multi‐omics technologies could reveal the miRNA‐mediated regulatory networks from multiple levels and layers, with the aim of precisely elucidating the BPs and underlying mechanisms involved in the pathogenesis of WD.

## 5. Summary

In summary, this study not only delineated the differential expression profile of serum exosomal miRNAs in individuals with WD, but more importantly, employed an integrated bioinformatic analysis encompassing GO, KEGG, Reactome, and DO databases. This comprehensive approach systematically revealed how these DE‐miRNAs potentially disrupt critical cellular processes—including ubiquitin‐mediated protein homeostasis, GPCR signaling cascades, Hippo pathway regulation, transcriptional control, and RNA splicing—thereby establishing a complex network underlying liver pathology, neurological dysfunction, and heightened systemic tumor susceptibility. These findings provide a novel conceptual framework for understanding the multisystem pathophysiology of WD and lay a robust theoretical foundation for developing exosomal miRNA‐based diagnostic biomarkers and therapeutic targets.

## Ethics Statement

The protocol of this clinical study is in full compliance with the ethical principles of the Declaration of Helsinki, as well as good clinical practice guidelines and applicable local regulatory requirements. This study was approved by The Institutional Review Committee of The First Affiliated Hospital of Anhui University of Traditional Chinese Medicine.

## Consent

The authors have nothing to report.

## Conflicts of Interest

The authors declare no conflicts of interest.

## Author Contributions

**Hong Chen:** formal analysis, writing – review and editing. **Xie Wang:** writing – original draft methodology. **Yue Pu:** formal analysis. **Ying Ma and Hao Ye**: visualization. **Juan Zhang:** supervision, fuding acquisition and review. Hong Chen and Xie Wang contributed equally to this manuscript.

## Funding

The present study was supported by the National Natural Science Foundation of China (Grant 82274493), Scientific Research Project of Higher Education Institutions in Anhui Province (Grant 2023AH050791), Outstanding Project of Jianghuai Talent Training Program in Anhui Province in 2023 (Anhui Organization Office (2024) No.18), High level Inheritance Talent Support Project for Traditional Chinese Medicine in Anhui Province (Anhui Traditional Chinese Medicine Development Secret (2024) No.1), the Foundation of Anhui Provincial Key Laboratory of Chinese Medicinal Formula (Grant 2025AKLCMF07), and Exploratory Research Project at Anhui University of Traditional Chinese Medicine (Grant AHUCM2024TS094).

## Data Availability

The datasets generated and/or analyzed during the current study are available from the corresponding author on reasonable request.
